# A cross-sectional study of owner-reported health in Canadian and American cats fed meat- and plant-based diets

**DOI:** 10.1186/s12917-021-02754-8

**Published:** 2021-01-28

**Authors:** Sarah A. S. Dodd, Cate Dewey, Deep Khosa, Adronie Verbrugghe

**Affiliations:** 1 Department of Population Medicine, Ontario Veterinary College, University of Guelph, 50 Stone Rd. E, Guelph, Ontario N1G 2W1 Canada; 2 Department of Clinical Studies, Ontario Veterinary College, University of Guelph, 50 Stone Rd. E, Guelph, Ontario N1G 2W1 Canada

**Keywords:** Alternative pet diet, Feline nutrition, Health perception, Pet feeding practices, Pet owner survey, Vegan cat

## Abstract

**Background:**

Cats, being obligate carnivores, have unique dietary requirements for nutrients most commonly found in dietary ingredients of animal origin. As such, feeding a diet devoid of animal-derived ingredients has been postulated as a possible cause of nutrient imbalances and adverse health outcomes. A small proportion of cat owners feed strictly plant-based diets to the cats in their care, yet the health and wellness of cats fed these diets has not been well documented.

**Results:**

A total of 1325 questionnaires were complete enough for inclusion. The only exclusion criterion was failure to answer all questions. Most cats, 65% (667/1026), represented in the survey were fed a meat-based diet and 18.2% (187/1026) were fed a plant-based diet, with the rest fed either a combination of plant-based with meat-based (69/1026, 6.7%) or indeterminable (103/1026, 10%). Cat age ranged from 4 months to 23 years, with a median of 7 years, and was not associated with diet type. No differences in reported lifespan were detected between diet types. Fewer cats fed plant-based diets reported to have gastrointestinal and hepatic disorders. Cats fed plant-based diets were reported to have more ideal body condition scores than cats fed a meat-based diet. More owners of cats fed plant-based diets reported their cat to be in very good health.

**Conclusions:**

Cat owner perception of the health and wellness of cats does not appear to be adversely affected by being fed a plant-based diet. Contrary to expectations, owners perceived no body system or disorder to be at particular risk when feeding a plant-based diet to cats. This study collected information from cat owners and is subject to bias, as well as methodological limitations. Further research is warranted to determine if these results are replicable in a prospective investigation.

**Supplementary Information:**

The online version contains supplementary material available at 10.1186/s12917-021-02754-8.

## Background

The domestic cat, *Felis catus*, is a small mammal of the order Carnivora considered to be an obligate carnivore, based on their evolutionary anatomical, physiological and metabolic adaptations to a diet exclusively comprised of prey [1, 2]. As a consequence, cats have unique nutritional adaptations resulting in particular dietary requirements [3, 4]. Briefly, in comparison to their omnivorous counterpart, the domestic dog, the cat’s requirement for total protein is higher, and they require dietary provision of taurine, long-chain polyunsaturated fatty acids and vitamin A [1, 5–8]. Protein content and quality, in terms of amino acid digestibility, bioavailability and balance, is typically higher in animal-derived as opposed to plant-derived ingredients [9]. Moreover, taurine, arachidonic acid, eicosapentaenoic acid (EPA), docosahexaenoic acid (DHA), and vitamin A are predominantly or exclusively found in animal tissues [10, 11]. Nevertheless, plant-based [PB] diets (diets entirely devoid of animal-derived ingredients, aka vegan diets) exist commercially and are marketed for feeding to domestic cats.

Associations between nutrition and health are known regarding domestic cats. Not only do cats require a diet providing balanced nutrition in order to avoid adverse health outcomes, certain disease states are also known to be associated with imbalances or inappropriate provision of particular nutrients [12–16]. Considering this, the type of diet a cat is fed may influence their day-to-day health, disease status, and even their longevity. As obligate carnivores, it has long been considered that cats require a diet that contains animal-derived ingredients to provide the essential nutrients they require, and the implications of feeding PB diets to cats are yet to be well described. However, nearly 1% of all cat owners, and 10% of vegan cat owners, choose to feed an entirely PB diet to their cat [17].

Previous publications regarding feeding of PB diets to carnivorous cats have focused either on the content of some essential nutrients in PB cat food or on measurement of some indicators of nutrient status in the blood of cats fed PB diets for at least 1 year [18–21]. Results from these studies have varied, with dietary nutrient insufficiencies documented [18, 19, 21], but no adverse health or nutritional outcomes detected [20, 21]. Nevertheless, it has been suggested that feeding PB diets to cats may predispose them to health disorders, including lower urinary tract diseases [21, 22]. Other studies have investigated the motivations and attitudes of cat owners towards feeding their cat, finding that meat-abstainers were the only ones who fed their cats PB diets, and that their impetus to do so was based largely on ethics or morality [17, 20, 23]. Currently, only one study investigating owner perception of health in cats fed PB has been published [20]. The objective of this study was to survey a wide sample of cat owners in order to describe beliefs and practices regarding cat health and nutrition and compare between owners of cats fed PB or meat-based [MB] diets. It was hypothesized that resources used for feline nutrition and factors influencing dietary decisions would differ between owners feeding cats PB or MB diets and that there would be a higher prevalence of health disorders among cats fed PB diets. In particular, it was hypothesized that more cats fed PB diets would be reported to have lower urinary tract disorders as compared to cats fed MB diets.

## Results

### Response rate and demographic information

A total of 1325 questionnaires were voluntarily undertaken and included for analysis, responses were excluded if they failed to answer all questions in the questionnaire. Partially completed surveys were included, thus the number of responses for each question varied as a result of non-responses to individual questions. Demographic data of respondents are shown in Supplementary Table S1. Post-hoc power calculations confirmed the sample size was adequate to attain statistical significance for comparison of the number of health disorders, specific health disorders (GI and hepatic) and lifespan between cats fed PB and MB diets.

The proportion of respondents keeping cat(s) only (653/1325 49%) did not differ from those keeping dog(s) and cat(s) (672/1325, 51%). The median number of cats kept per respondent was two (range 1–18). Most cats were acquired from shelters, rescues or veterinarians (664/1241, 54%), followed by tamed stray, feral or found cats (214/1241, 17%) or inherited or gifted from friends and family (167/1241, 13%). Less commonly, cats were purchased online (64/1241, 5.2%), from backyard breeders, farms, or homebred (59/1241, 4.8%), from registered breeders (44/1241, 3.5%) or from pet stores (29/1241, 2.3%). Cats were acquired by respondents at kittenhood (645/1241, 55%) or later in their life (538/1241, 45%). Cats had been kept by the respondents for less than 1 to 25 years (mean 6.0 years, std. dev. 4.85).

### Cat characteristics

Cat characteristics are shown in Table 1, grouped by diet type. There was a significant association between diet and breed type, with more MB cats being domestic longhairs (66/656, 10% MB; 8/182, 4.4% PB), while more PB cats were mix breeds (58/656, 8.8% MB; 29/182, 16% PB), or their breed unknown (46/656, 7.0% MB; 25/182, 14% PB) (*P* = 0.004). No significant differences in breed types were detected between PB+MB and PB or MB. Mean cat age was reported to be 7.5 years (std. dev. 4.85). There was no association between cat age and diet. Most cats lived indoors only (749/1246, 60%), many had unlimited outdoor access (373/1246, 30%), some had limited or controlled outdoor access (113/1246, 9.1%), and few lived outdoors exclusively (11/1246, 0.88%). The majority of respondents indicated that their cats did not hunt prey (949/1241, 76%). Some respondents recognized that their cats could hunt but they did not believe that they did so (91/1241, 7.3%) and less than a quarter acknowledged that their cat did hunt prey (201/1241, 16%). Though there was no association between access to the outdoors and diet, significantly (*P* = 0.005) more PB cats were reported to not to hunt (129/186, 69%) or to have the ability to hunt but not do so (23/186, 12%). Of the cats fed a PB diet, 35% (65/187) were reported unlimited access to the outdoors, suggesting their diet could be supplemented with hunted prey to some degree (PB+MB/H).

**Table 1:** Characteristics of cats as reported by participants in the “Pet Health and Wellbeing” survey

	MB (*n* = 667)	PB (*n* = 187)	PB+MB (*n* = 69)
Sex			
Male (*n* = 611)	321 (48%)	94 (50%)	34 (49%)
Female (*n* = 653)	342 (51%)	93 (50%)	35 (51%)
Sex status			
Intact (*n* = 41)	17 (3%)	10 (5%)	3 (4%)
Desexed (*n* = 1223)	646 (97%)	177 (95%)	66 (96%)
Breed type			
DSH (*n* = 660)	362 (54%)	93 (50%)	36 (52%)
DMH (*n* = 60)	35 (5%)	6 (3%)	5 (7%)
DLH (*n* = 95)	66 (10%)	8 (4%)	1 (1%)
Asian (*n* = 50)	32 (5%)	8 (4%)	2 (3%)
American (*n* = 35)	21 (3%)	4 (2%)	1 (1%)
European (*n* = 58)	34 (5%)	8 (4%)	2 (3%)
Other (*n =* 9)	2 (0%)	1 (0%)	0 (0%)
Mix (*n* = 134)	58 (9%)	29 (16%)	9 (13%)
Unknown (*n* = 131)	46 (7%)	25 (14%)	11 (16%)
Age (years)			
Less than 1	29 (4%)	7 (4%)	3 (4%)
1–2	92 (14%)	28 (15%)	6 (9%)
3–4	105 (16%)	24 (13%)	6 (9%)
5–6	83 (12%)	34 (18%)	14 (20%)
7–8	90 (13%)	24 (13%)	6 (9%)
9–10	65 (10%)	19 (10%)	12 (17%)
11–12	61 (9%)	21 (11%)	5 (7%)
13–14	41 (6%)	18 (10%)	5 (7%)
15–16	40 (6%)	6 (3%)	6 (9%)
17–18	16 (2%)	6 (3%)	2 (3%)
19–20	9 (1%)	2 (1%)	0 (0%)
Greater than 20	1 (0%)	0 (0%)	0 (0%)

Numbers of cats per category may not add up to total due to non-responders and indeterminable diet type. Thirty-four specific breeds were reported, including: Abyssinian, American bobtail, Balinese, Bengal, Birman, Bombay, British shorthair, Burmese, Chantilly, Chartruex, Chaussie, domestic shorthair (DSH), domestic medium hair (DMH), domestic longhair (DLH), Havana brown, Himalayan, Korat, Maine coon, Manx, Norwegian forest cat, Oriental, Persian, Ragdoll, Rex, Russian blue, Siamese, Siberian, Snowshoe, Somali, Sphynx, Tonkinese, Toyger, Turkish angora, Turkish van

### Cat diet

Diet was described by 1026/1325 respondents. Most cats were fed a MB diet (667/1026, 65%), less than a quarter were fed strictly PB (187/1026, 18%), and a small proportion were fed a combination of PB and MB (PB+MB, 69/1026, 6.7%). Diet type was indeterminable for 10% (103/1026) of cats. For cat health and wellness comparative analyses, cats that were fed PB but had access to the outdoors and ability to hunt were re-classified as PB+MB/H (139/1026, 14%).

Cats had been reportedly fed their current diet their whole life (534/1034 52%) or had changed from at least one previous diet to their current diet (500/1034, 48%). Cats had been fed their current diet for a mean of 3.8 years (std. dev. 3.95). There was no difference in duration of feeding the current diet between cats fed MB (mean 3.6 years, std. dev. 3.69) PB (mean 3.6 years std. dev. 4.38) or PB+MB (mean 3.0, std. dev. 3.27) diets. Less than half of cats received treats (386/1026, 38%) or table scraps (220/1026, 21%) in addition to their main diet. More cats fed MB (308/667, 46%) than PB (29/187, 16%) or PB+MB (16/69, 23%) received treats (*P* < 0.001), though more cats fed PB (63/187, 34%) than MB (128/667, 19%) received table foods (*P* < 0.001). No significant differences in feeding of table foods were detected between PB+MB and PB or MB. Significant differences in the feeding of table foods was evident between PB and MB cats (*P* < 0.001). The most common table foods fed to MB cats were meat (66/667, 9.9%), dairy or eggs (64/667, 9.6%) and fruits or vegetables (20/667, 3.0%). The most common table foods fed to PB cats were fruits or vegetables (34/187, 18%), nuts, seeds, legumes or grains (21/187, 11%), and plant-based meat alternatives (16/187, 8.6%). Cats fed PB+MB were most commonly offered dairy or eggs (10/69, 14%), fruits or vegetables (7/69, 10%), or meat (5/69, 7.3%). Supplements were fed to less than a quarter of cats (*n* = 193/1026, 19%), more so to those fed PB (75/187, 40%) than PB+MB (17/69, 24%) or MB (91/667, 14%) (*P* < 0.001). Not only were the number of cats offered supplements different between diet groups, but also the types of supplements given. Overall, ‘functional foods’ such as cranberry powder, coconut oil and yeast were the most common supplements offered (77/923, 8.3%), followed by fibre, pre- or probiotics (38/923, 4.1%) and vitamins and/or minerals (36/923, 3.9%). Cats fed PB were given more ‘functional foods’ (55/187, 29%), supplements marketed for specific disorders (14/187, 7.5%), and digestive enzymes (8/187, 4.3%), than cats fed PB+MB (functional foods 6/69, 8.7%; specific disorders 2/69, 2.9%; enzymes (0/69, 0%) or MB (functional foods 16/667, 2.4%; specific disorders 19/667, 3.9%; enzymes 9/667, 1.4%). There were no differences between diet groups and the feeding of marine derived fatty acids, multivitamin/minerals, fibre or pre/probiotics, amino acids, cannabis products, or herbs.

### Cat health and wellness

Cat BCS ranged from 1 to 9. Most cats were reported to be in ideal condition (677/1233, 55%), and more cats were overweight (405/1233, 33%) than underweight (151/1233, 12%); median BCS was 5. Cat BCS differed significantly between cats fed PB and MB (*P* = 0.13). More owners of cats fed strictly plant-based reported their cat to have an ideal BCS (83/117, 71%) and fewer reported overweight (23/117, 20%), as compared to cats fed MB (358/666, 54% ideal; 226/666, 34% overweight). No difference was detected for PB+MB/H compared to PB (*P* = 0.196) or MB (*P* = 0.635).

Based on FS, most cats were reported to have normal faeces (920/1047, 88%), few were constipated (33/1047, 3.2%) or had soft to diarrhoeic faeces (94/1047, 9.0%); median FS was 2 (range 1–7) on a 1–7 scale, with 3–4 being ideal, 1 being constipation and 7 being diarrhoea. There were no significant differences in FS between cats fed MB, PB or PB+MB/H diets.

Health disorders were reported by the respondent and categorized by body system or systemic disorders as appropriate. The number of health disorders reported per cat ranged from 0 to 6 (median 0), and half of all cats (628/1208, 52%) were reported to have no health disorders. Prevalence of the most commonly reported health disorders are shown in Table 2. A negative binomial model was designed to determine if the number of health disorders a cat had was associated with diet. With cat age and sex included in the model, the number of disorders per cat differed significantly based on diet, after controlling for demographic and lifestyle variables.

**Table 2:** Prevalence of feline health disorders as reported by participants in the “Pet Health and Wellbeing” survey, with comparison between cats fed different diets

Health disorder	Total		MB		PB		PB+MB/H	
	*n* = 1208	%	*n* = 667	%	*n* = 117	%	*n* = 139	%
Cardiac disease	26	2.2	17	2.6	2	1.7	20	0
Dental disease	208	17	131	20	21	18	16	11.5
Dermatopathy	137	11	82	12	12	10.3	13	9.4
Endocrinopathy	38	3.2	24	3.6	3	2.6	3	2.2
GI and hepatic diseases	126	10	90	13	3	2.6	11	7.9
Lower urinary tract disease	132	11	74	11	13	11	12	13
Neoplasia	21	1.7	10	1.5	2	1.7	4	2.9
Neurological	19	1.6	15	2.3	3	2.6	0	0
Obesity	100	8	64	9.6	7	6.0	9	6.5
Ocular disorders	57	5	39	5.9	4	3.4	1	0.7
Renal disease	42	3	31	4.7	1	0.9	2	1.4

*MB* meat-based, *PB* plant-based, *PB+MB/H* plant-based with animal-derived treats/snacks/supplements and/or ability to hunt

Numbers of cats per category may not add up to total due to non-responders and indeterminable diet type. No statistically significant differences were detected

Cat age (Coef. 0.09, 95% CI 0.070–0.100, *P* < 0.001) and male sex (Coef. 0.16, 95% CI 0.004–0.308, *P* = 0.044) were associated with increased number of disorders, while PB (Coef. -0.40, 95% CI -0.641 – 0.155, *P* = 0.001) and PB+MB/H (Coef. -0.33, 95% CI -0.589 - -0.075, *P* = 0.011), as compared to MB, were associated with fewer disorders. The relationships between diet and individual health disorders were investigated using logistic regression models, results are shown in Table 3. Overall, after controlling for age, sex, breed type, and body conditions score, diet was significantly associated only with dental, GI and hepatic, and ocular disorders. Age was associated with most health disorders.

**Table 3:** Results from multivariable logistic regression models of associations between reporting of health disorders and cat diet type, with confounders (age, breed type, sex, body condition score) controlled for

Health Disorder	Variable	Odds Ratio	95% CI	*P*-value
Cardiac	Age	1.15	1.045–1.258	0.004
	Breed type, DLH	3.58	1.006–12.700	0.003
	Diet, PB	0.69	0.152–3.162	0.636
	Diet, PB+MB/H	–		
Dental	Age	1.11	1.070–1.152	< 0.001
	Diet, PB	0.84	0.495–1.434	0.528
	Diet, PB+MB/H	0.51	0.287–0.891	0.018
Dermatological	Age	1.08	1.038–1.129	< 0.001
	Diet, PB	0.79	0.415–1.512	0.480
	Diet, PB+MB/H	0.64	0.337 0 1.215	0.172
Endocrine	Age	1.37	1.234–1.519	< 0.001
	BCS, 3	0.13	0.030–0.561	< 0.001
	BCS, 5	0.07	0.018–0.272	< 0.001
	BCS, 7	0.07	0.015–0.315	0.001
	BCS, 9	0.05	0.008–0.359	0.003
	Diet, PB	0.57	0.156–2.113	0.248
	Diet, PB+MB/H	0.47	0.131–0.356	0.404
GI and hepatic	Age	1.09	1.049–1.140	< 0.001
	Diet, PB	0.16	0.051–0.530	0.003
	Diet, PB+MB/H	0.53	0.273–1.028	0.060
Lower urinary tract	Age	1.10	1.057–1.152	< 0.001
	Sex, male	2.88	1.836–4.518	< 0.001
	Diet, PB	0.96	0.508–1.827	0.908
	Diet, PB+MB/H	1.20	0.678–2.127	0.534
Neoplasia	Age	1.32	1.180–1.487	< 0.001
	Diet, PB	1.10	0.228–5.305	0.907
	Diet, PB+MB/H	1.83	0.543–6.194	0.329
Neurological	Diet, PB	1.14	0.326–4.014	0.834
	Diet, PB+MB/H	–		
Obesity	BCS, 9	8.50	1.064–67.940	0.041
	Diet, PB	0.84	0.360–1.977	0.695
	Diet, PB+MB/H	0.74	0.347–1.582	0.438
Ocular	Breed type, mix	3.66	1.609–8.321	0.003
	Diet, PB	0.64	0.218–1.849	0.406
	Diet, PB+MB/H	0.11	0.015–0.923	0.027
Renal	Age	1.30	1.196–1.411	< 0.001
	Diet, PB	0.16	0.021–1.250	0.081
	Diet, PB+MB/H	0.27	0.061–1.168	0.080

Referent categories: Breed type = domestic shorthair, Diet = meat-based, BCS = 1, sex = female

*DLH* domestic longhair, *PB* plant-based, *PB+MB/H* plant-based with animal-derived treats/snacks/supplements and/or ability to hunt, *BCS* body condition score. Odds ratios for categories with insufficient numbers could not be computed (−)

**Fig. 1:**
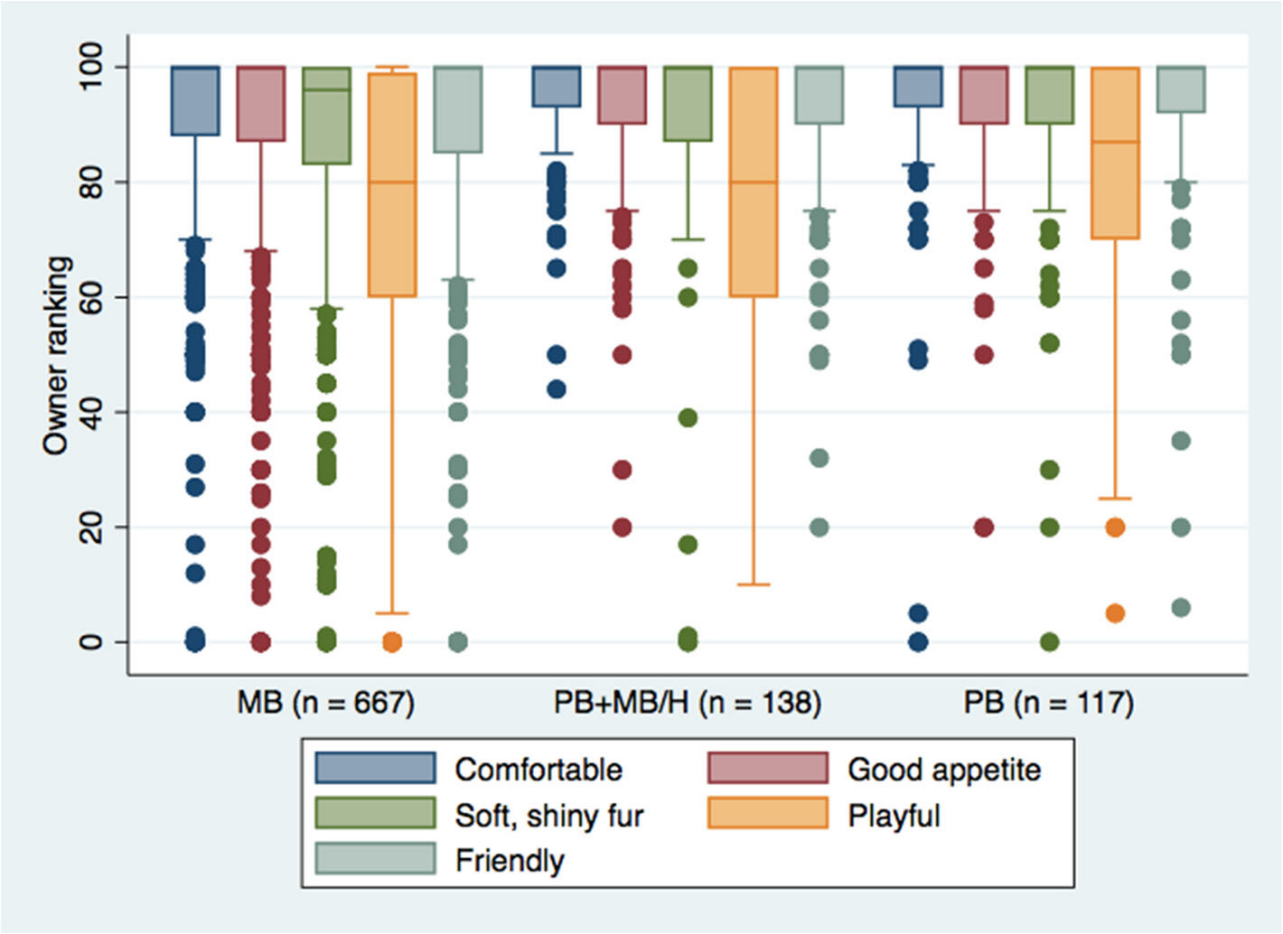
Owner ranking of positive cat wellness indicators based on visual sliding scale ranging from 0 (lowest) to 100 (highest), with comparison between cats fed different diets. *n* = 1147. MB = meat-based, PB = plant-based, PB+MB/H = plant-based with animal-derived treats/snacks/supplements and/or ability to hunt

**Fig. 2:**
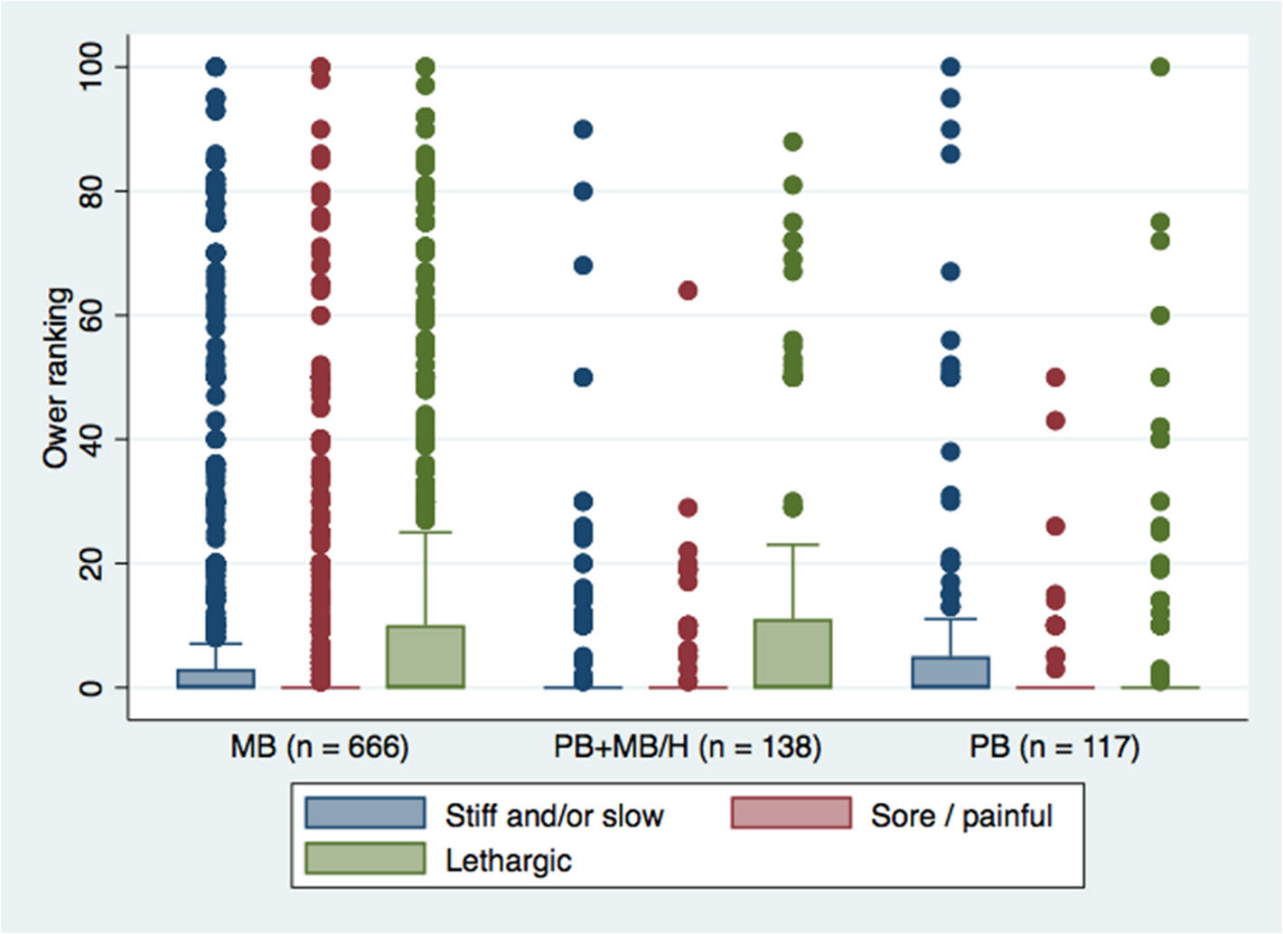
Owner ranking of negative cat wellness indicators based on visual sliding scale ranging from 0 (lowest) to 100 (highest), with comparison between cats fed different diets. *n* = 1145. MB = meat-based, PB = plant-based, PB+MB/H = plant-based with animal-derived treats/snacks/supplements and/or ability to hunt

Respondents’ perception of their cat’s health, from poor to very good, was predominantly very good (813/1206, 67%). Few respondents indicated their cat was in fair (47/1206, 3.9%) or poor (11/1206, 0.91%) health. An ordered logistic regression model was designed to determine if owner perception of cat health was associated with diet. Increasing cat age (Odds Ratio [OR] 0.84, 95% CI 0.808–0.863, *P* < 0.001) and male sex (OR 0.71, 95% CI 0.523–0.976, *P* = 0.035) were associated with lower odds of ranking cat health as very good. Conversely, a BCS of 5 (OR 7.72, 95% CI 2.67–22.29, *P* < 0.01) or 7 (OR 4.55, 95% CI 1.552–13.337, *P* = 0.006) as compared to 1, and feeding a PB (OR 1.99, 95% CI 1.194–3.329, *P* = 0.008) or PB+MB/H (OR 2.19, 95% CI 1.360–3.511, *P* = 0.001), as compared to MB, were associated with greater odds of ranking cat health to be very good, after controlling for cat demographics. Respondents also ranked their cat’s wellness based on seven Likert scale items (Table 4), and eight visual scales ranging from 0 (lowest) to 100 (highest) (Figs. 1 and 2).

**Table 4:** Responses to seven Likert scale questions asking respondents to rank indices of cat wellness, with comparison between cats fed different diets

Wellness Indicator		Total		MB		PB+MB/H		PB	
		*n* = 1025	%	*n* = 665	%	*n* = 138	%	*n* = 117	%
Frequency of vomiting	Not at all	629	61	395	59	100	72	76	65
	A little	369	36	256	39	37	27	38	32
	Quite a bit	24	2.3	14	2.1	1	0.7	3	2.6
Frequency of inactivity	Not at all	783	76	492	74	115	83	103	88
	A little	208	20	147	22	19	14	13	11
	Quite a bit	34	3.3	28	4.2	4	2.9	1	0.9
Happy appearance	Not at all	3	0.3	1	0.2	0	0	1	0.9
	A little	31	3.0	23	3.5	1	0.7	3	2.6
	A moderate amount	316	31	206	31	39	28	34	29
	A great deal	675	66	437	66	98	71	79	68
Distress vocalization	Not at all	826	81	530	80	112	82	99	85
	A little	174	17	119	18	22	16	17	15
	A moderate amount	19	1.9	14	2.1	2	1.5	1	0.9
	A great deal	4	0.4	3	0.5	1	0.7	0	0
Demonstration of affection	Not at all	7	0.7	6	0.9	0	0	0	0
	A little	54	5.3	26	3.9	6	4.4	8	6.8
	A moderate amount	309	30	217	33	38	28	24	21
	A great deal	654	64	417	63	94	68	85	73
Contact avoidance	Not at all	735	72	762	70	114	82	92	79
	A little	236	23	170	26	18	13	23	20
	A moderate amount	43	4.2	28	4.2	5	3.6	1	0.9
	A great deal	7	0.7	4	0.6	1	0.7	1	0.9
Curious behaviour	Not at all	10	1.0	8	1.2	2	1.5	0	0
	A little	94	9.2	64	9.6	8	5.9	7	6.0
	A moderate amount	417	41	268	40	58	42	52	44
	A great deal	502	49	326	49	69	50	58	50

*MB* meat-based, *PB* plant-based, *PB+MB/H* plant-based with animal-derived treats/snacks/supplements and/or ability to hunt

**Fig. 3:**
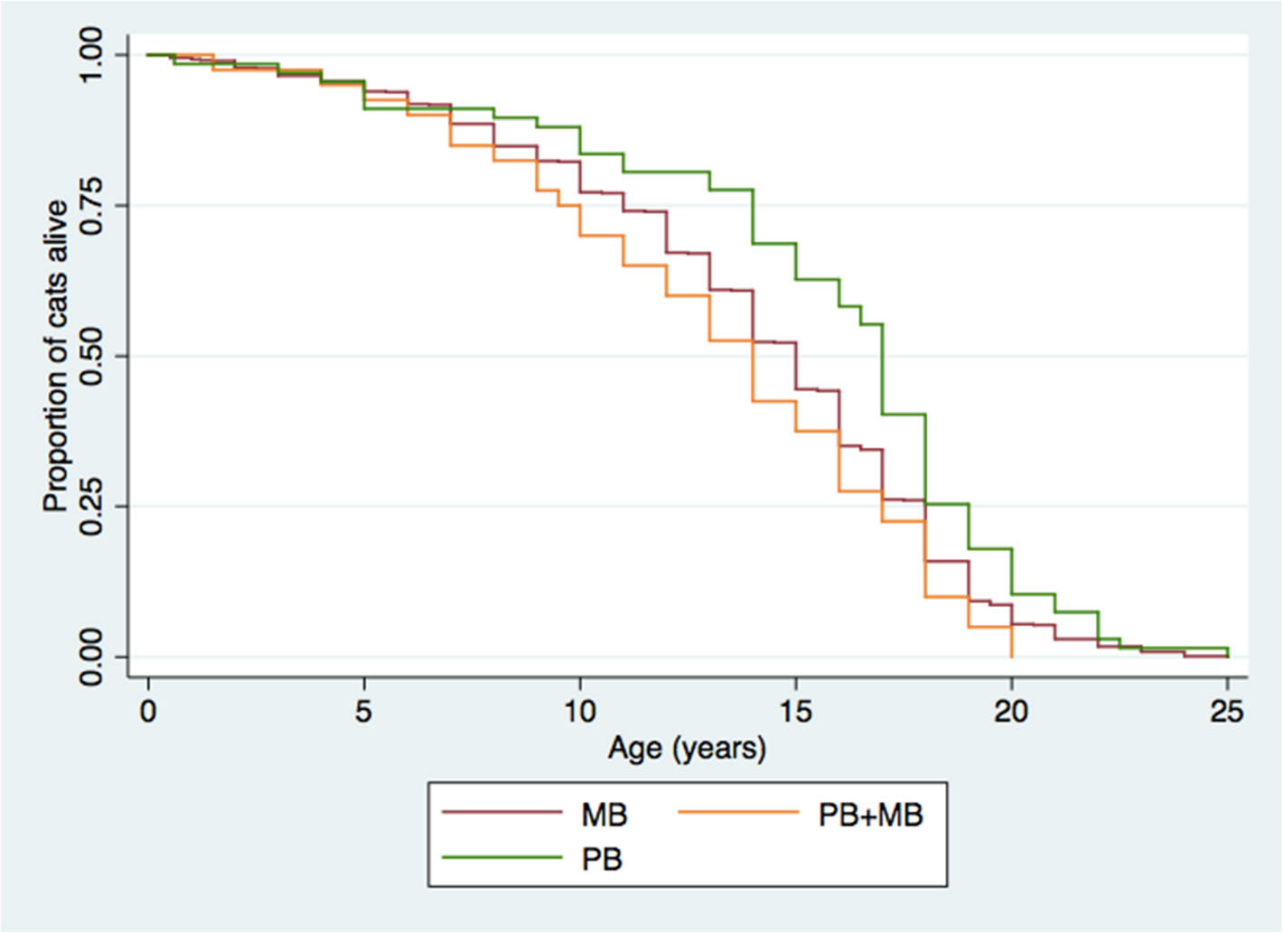
Kaplan Meier survival function of previous cats as reported by participants in the “Pet Health and Wellbeing” survey, with comparison between cats fed different diets. No statistically significant differences were detected by log-rank test, *P* = 0.192. MB = meat-based (*n* = 807), PB = plant-based (*n* = 67), PB+MB = plant-based with animal-derived treats/snacks/supplements (*n* = 40)

Lifespan was indicated when respondents were asked about previous cats. Mean lifespan was reported to be 14 years (std. dev. 4.88). Previous cats were fed MB (841/1161, 72%), PB (77/1161, 6.6%), a combination of MB and PB (41/1161, 3.5%) or indeterminable (258/1161, 22%). There was no significant difference in reported lifespan based on diet detected by log rank test (Fig. 3).

### Diet changes

Owners typically fed their current cat the same type of diet (i.e. PB or MB) as they fed their previous cat(s). Of the owners who fed their previous cat(s) a PB diet, only 4.0% (2/50) added some animal products and 2.0% (1/50) changed to a MB diet for their current cat. Of the owners who fed a PB+MB to their previous cat(s), most continued to feed PB+MB diet (10/25, 40%), while relatively even proportions changed to a completely PB (6/25, 24%) or MB (8/25, 32%) diet for their current cat. Of the owners who previously fed their cat(s) a MB diet, the majority of owners continued to feed this diet type to their current cat (514/637, 81%), though more changed to an entirely PB diet (73/637, 11%) than a PB+MB (31/637, 4.9%).

### Diet selection

Cat food was predominantly chosen based on whether or not it was complete and balanced (775/1106, 70%). Table 5 shows the most common criteria reported for cat food selection. Of the 14% of owners (156/1106) who reported concern regarding specific ingredients in their cat’s diet, significant differences were found between feeders of MB, PB and PB+MB diets. Owners feeding their cats MB most commonly reported a desire to avoid ingredients like by-products, grains, ‘fillers’ or additives (4/187, 2.1% PB; 2/69, 2.9% PB+MB; 60/667, 9.0% MB; *P* = 0.002), and for a diet to contain animal ingredients high on the list of ingredients (0/187, 0% PB; 0/69, 0% PB+MB; 30/667, 4.5% MB; *P* = 0.008). More owners feeding their cats a PB diet were wanted to see inclusion of particular nutritional additives (e.g. specific vitamins) (12/187, 6.4%) as compared to PB+MB (0/69, 0%) or MB (8/667, 1.2%) (*P* < 0.001). There were no differences between diet groups and the desire to avoid specific plant-derived ingredients (e.g. corn, soy). Respondents indicated the resource(s) they used for information about feline nutrition. The common sources of information and significant differences between diet groups are shown in Table 6. Veterinary professionals were the most common (707/1022, 64%), followed by internet and social media (681/1022, 62%). Of the respondents who indicated using the internet as a resource for feline nutrition, over half (396/681, 58%), also used a veterinarian.

**Table 5:** Factors influencing pet food purchasing as reported by participants in the “Pet Health and Wellbeing” survey, with comparison between owners of cats fed different diets

Criteria	Total		MB		PB		PB+MB	
	*n* = 1025	%	*n* = 667	%	*n* = 187	%	*n* = 69	%
Complete and balanced	775	70	462	69^a^	151	81^b^	51	74^a,b^
Convenience	321	29	198	30	51	27	23	33
Human grade	150	14	84	13^a^	39	21^b^	7	10^a^
Natural/organic/holistic	327	30	152	23^a^	95	51^b^	36	52^b^
Palatability	316	29	218	33^a^	47	25^b^	16	23^b^
Price	314	28	204	31^a^	42	22^b^	22	32^a^
Skin/coat/hairball health	272	25	174	26	44	24	11	16
Specific ingredients	156	14	115	17^a^	21	11^b^	4	5.8^b^
Stool quality	133	12	83	12	25	13	8	12
Therapeutic/vet recommended	186	17	144	22^a^	4	2^b^	7	10^c^

*MB* meat-based, *PB* plant-based, *PB+MB* plant-based with animal-derived treats/snacks/supplements

Superscript characters denote significant (*P* < 0.05) differences between the diet categories

Values may add up to > 100% since respondents could indicate that they selected pet food based on more than one criterion and numbers of cats per category may not add up to total due to non-responders and indeterminable diet type

**Table 6:** Resources used to acquire feline nutrition information as reported by participants in the “Pet Health and Wellbeing” survey, with comparison between owners of cats fed different diets

Source of information	Total		MB		PB		PB+MB	
	*n* = 1022	%	*n* = 665	%	*n* = 187	%	*n* = 69	%
Book, pamphlet or printed resource	174	16	95	14^a^	43	23^b^	16	23^b^
Breeder or shelter	20	2	17	2.6	1	0.5	0	0
Discussion group	272	25	145	22^a^	67	36^b^	24	35^b^
Friends and/or family	186	17	100	15^a^	30	16^a^	18	26^b^
Internet and social media	681	62	361	54^a^	164	88^b^	53	76^c^
Manufacturer	21	2	13	2.0	3	1.6	3	4.4
Pet store or vendor	229	21	143	22^a^	26	14^b^	17	25^a^
School or courses	39	4	31	4.7	2	1.1	0	0
Veterinary technician, clinician, specialist, student	707	64	465	70^a^	87	47^b^	38	55^b^

*MB* meat-based, *PB* plant-based, *PB+MB* plant-based with animal-derived treats/snacks/supplements

Superscript characters denote significant (*P* < 0.05) differences between the diet categories

Values may add up to > 100% since respondents could indicate that they sought information from more than one source and numbers of cats per category may not add up to total due to non-responders and indeterminable diet type

## Discussion

### Response rate and demographic information

Participants in this study typically represented the demographics of previously reported pet food and pet health survey respondents [17, 24, 25], supporting the likelihood that the sampled population was consistent with other publications in the field. However, the sampling methodology was not random, but invited cat owners to self-select to participate in the study. This may have biased the types of owners and cats represented by the study. The sampling included owners of both dogs and cats, not just cats exclusively, though only responses relating to cats were included in these analyses. In the USA, almost half (47%) of cat owners also own dogs [26], making the input of these owners as relevant as the input of cat-exclusive owners.

### Cat characteristics

Cat keeping characteristics, including number of cats, cat acquisition, and indoor/outdoor lifestyle, as well as cat characteristics such as median age, breed, sex and sex status, were similar to previous publications in Canada, the USA and worldwide [27–29], supporting the likelihood that the sampled population was representative of the general cat population in the regions of interest.

### Cat diet, health and wellness

Despite their carnivorous physiology and metabolism, approximately 1% of cat owners feed a PB diet to their cat [17]. A previous study evaluated the attitudes of cat owners and compare between cats fed vegetarian and conventional MB diets [20]. In that study, over three quarters of owners feeding their cats a vegetarian diet considered the diet to be beneficial for decreased risk of cancer, healthy coat, longevity, weight control and/or reduced risk of allergies, and just over 10 % considered there to be no benefit. When queried regarding the risks of vegetarian diets, over three quarters reported concern for retinal atrophy, taurine deficiency, lower urinary tract disease, protein deficiency, dilated cardiomyopathy, while less than a quarter considered there to be no risk to health. In a recent investigation, though nearly two-thirds of owners feeding their pet a PB diet reported concern for the diet providing incomplete nutrition, though less than half reported a concern regarding risk to health [17]. This apparently positive perception of PB diets previously documented was supported by the findings of the present study, where more owners of cats fed PB reported their cat to be in very good, as opposed to good, overall health. Additionally, owners of cats fed PB diets reported fewer health disorders in their cats. In previous evaluations of the health of cats fed PB diets, no adverse health outcomes attributable to diet were found, and most parameters measured were within the normal reference range or above minimum thresholds [20, 21].

Among veterinarians, animal nutritionists and veterinary nutritionists, it is generally considered contraindicated to feed a strictly PB diet to cats [19, 30, 31]. Considering this, it was predicted that feeding a strictly PB diet to cats may result in poorer health and/or wellness. However, this was not supported by the findings of this study. Owner perception of health and wellness, as reported by participants in the study, were largely comparable between cats fed MB and PB diets (Table 4, Figs. 1 and 2), and in some cases better for cats fed PB. PB diets have also been effectively used as growth and maintenance diets for other captive carnivores, including American alligators and carnivorous fish [32–35], suggesting that the maintenance of carnivorous cats using PB diets may not be as novel or unconventional as is commonly considered. It must be noted, however, that diets for animals intended for human consumption are typically designed to maximize some component of production and are not necessarily designed for optimal animal health or longevity. Nevertheless, longevity of cats, as reported by the study participants, did not vary between cats fed PB or MB diets, and was comparable to the recognized lifespan of domestic cats [36].

Specific concerns raised regarding PB diets for cats have typically been for the total protein content in comparison to feline requirements, risk of taurine deficiency and related disorders, and the carbohydrate content of the diet and risk of obesity and diabetes. Though total protein in commercial PB cat foods has been demonstrated to be sufficient, the amino acid profiles may be variable. In one study, five of six diets intended for feeding to cats failed to meet the recommended minimum value for one or more amino acids [18]. In the two canned products, taurine was below the recommended minimum. Thus, concern for taurine deficiency and protein malnutrition appears warranted. Nevertheless, to the authors’ knowledge, no case reports of protein malnutrition or taurine deficiency in cats fed PB diets have been published. Interestingly, despite the concern for amino acids, a common supplement in human PB nutrition, few cats were offered protein or amino acid supplementation. Though three times as many cats fed PB than MB received supplements in addition to their food, the supplements fed were most commonly ‘functional foods’ (30%), and those marketed for treatment or prevention of specific health disorders (7.5%), neither of which are likely to have contributed greatly to the nutritional value of the cats’ diet.

In addition to concerns for health disorders related to inappropriate protein and amino acid provision, concerns regarding the carbohydrate content of PB diets have also been raised. Carbohydrates have been suggested by some to contribute to feline obesity [37, 38]. The proportion of cats reported by the owner to be overweight in this study was in close agreement with the proportion reported to be overweight by the largest USA-based survey of pet owners [26]. Interestingly, BCS were reportedly more ideal and less overweight in cats fed PB than MB diets. The current body of evidence points towards imbalanced energy intake versus expenditure as being the predominant cause of obesity, as opposed to intake of any single macronutrient, though fat, being the most energy-dense and lipogenic nutrient, is of more concern than carbohydrates as a predisposing factor for feline obesity [39–41]. For a recent review of the role of carbohydrates in feline nutrition, see Verbrugghe and Hesta, 2017 [42]. It is possible that PB foods fed to cats may have lower dietary fat than MB diets, and likely that the higher fibre levels in plant ingredients versus meat ingredients may reduce energy density, help to regulate glucose tolerance and insulin sensitivity, and thus be protective against obesity, as has been demonstrated in humans [43–46]. Similarly, though diets with high levels of carbohydrates have been postulated to contribute to faecal abnormalities in cats [42, 47], with high levels of digestible carbohydrates and soluble fibres potentially causing diarrhoea and high levels of insoluble fibres potentially contributing to constipation, no difference in FS was attributable to a PB compared with a MB diet. Body and faecal condition are two health markers pet owners can easily keep track of at home. Similarly, hair condition, behaviour and activity levels are also commonly considered by pet owners and veterinarians alike to be general indicators of health.

Prevalence of health disorders in the overall study population were comparable to the reported prevalence in general populations as determined from veterinary visits [48, 49]. When total number of disorders was compared, significantly fewer were reported in cats fed PB compared to MB diets. When comparing individual disorders, reporting of GI and hepatic disorders was lower in cats fed PB as opposed to MB diets. No disorders were reportedly higher in cats fed PB. It is possible that PB diets confer some protection against these particular disorders, namely renal disorder and GI and hepatic disorders; indeed this has been demonstrated in humans [50–53]. However, in humans the health benefits of a PB diet have primarily been with respect to obesity, diabetes, cardiac and neoplastic disorders [54–58], and, unlike in humans, no statistically significant differences were reported in the prevalence of these disorders in cats. Of interest, the reported prevalence of disorders expected to be higher in cats fed PB diets, such as urinary tract disease [22], did not differ between diet groups in this study. At this time, to the authors’ knowledge, no cases of any adverse health outcome associated with PB diets in cats have been published, though a lack of evidence should not be interpreted as evidence of lack of risk. Nutrient deficiencies and imbalances may take many years to develop clinical signs, particularly in adult animals, and may go undetected.

### Diet changes

It is possible that upon diagnosis of a health disorder a cat’s diet is changed, most likely to a therapeutic diet. Considering no current therapeutic diets for cats are PB, it is possible that some cats diagnosed with a health disorder are changed from a PB to a MB diet, thus reducing the number of cats with health disorders in the PB category. Given the study design, this information was not able to be discerned. Interestingly, when comparing the diet of previous cats to current cats, few owners changed diet type, most continued to feed either PB or MB to current cats if that is what they fed their previous cat. Only a single owner who fed PB previously changed entirely to a MB diet, while about 10% of owners who previously fed MB changed entirely to a PB diet. Comparing diets of previous cats to current cats does not give any indication as to whether the diet of the current cat was changed in response to diagnosis of an adverse health disorder, but the lack of dietary change between cats may support a hypothesis that the incident of diet change from PB to MB may be low. Further research would be required to determine if this is indeed the case. The present data only indicates that cat owners who fed PB to previous cats continue to feed PB to new cats, while a proportion who fed MB to previous cats may adopt a PB diet for their new cat. This supports the suspicion that this trend is likely actively growing at this point in time, as has previously been hypothesized [17]. It is suspected that this may occur in conjunction with increasing proportions of people choosing to follow a vegan lifestyle for themselves, and not because of a perception of PB diets being healthier for cats. Previous research has demonstrated that a lack of concern for risk of negative impact on health was a determining factor for vegans feeding a PB to their pet, though a perception of improved health has not been reported [17]. This warrants further investigation as to what health benefits may be perceived to be associated with PB diets for cats.

### Diet selection

Of interest are the differences in the sources where cat owners sought information about feline nutrition and the criteria used to select a diet for their cat reported in the present study. Overall, veterinary professionals were equally represented with the internet and social media as being the resources used by the most cat owners, which is in good agreement with previous reports [59]. It is unclear if this referred to using a veterinary professional as well as a separate internet resource, or whether this indicated the use of a virtual veterinary resource, such as a veterinary blog or clinic website. However, when comparing between owners of cats fed PB or MB, significantly fewer owners feeding PB considered their veterinary team, instead relying much more heavily on the internet and social media, as well as discussion groups and books and printed resources to a lesser extent. This finding is not surprising, as it has been suggested that pet owners feeding PB diets may not feel comfortable discussing their feeding practice with their veterinarian and may not view their veterinarian as an informed resource for discussing PB nutrition [17]. This may be an issue not specific to PB feeding practices, per se, but to practices considered unconventional or alternative to the mainstream, as it has been reported that feeders of raw MB diets also show a decreased trust in veterinarians and seek nutritional information elsewhere [24]. A large risk of reliance on resources online or in print is the lack of quality control and critical evaluation these media undergo. While some websites, books, or other media may be useful resources with up-to-date and accurate information, it may not be apparent to the pet owner which are useful and which may be misleading and potentially harmful. For this reason, discussion of pet diet with the pet’s veterinarian is recommended. The criteria used to select pet food differed as well between feeders of MB or PB diets. In comparison to the owners feeding MB, those feeding PB had a greater concern for the diet being labelled complete and balanced, being a human grade food product, being marketed as natural, organic or holistic, and had less concern for the palatability of the diet or the presence or lack of specific ingredients. While human grade, natural, organic or holistic products are not recognized by many practitioners to be superior to their conventional counterparts, it is positive to note the increased awareness or concern of owners feeding PB regarding the labelling of a diet being complete and balanced. While a statement of nutritional adequacy in accordance with industry guidelines is not a guarantee that the product is indeed appropriate for the animal for which it is intended to be fed [60, 61], it is a benchmark by which pet foods are compared and is a minimum standard to ensure nutritional sufficiency.

### Limitations

The findings presented in this study must be interpreted with recognition of the inherent bias and limitation associated with the methodology. The sampling strategy employed allowed for self-selection into the study which likely introduces bias with respect to the nature of the participants. This sampling strategy was employed as the intention was to collect a large number of responses from broad sample of pet owners, representing the diversity within the pet owning population and avoiding targeting of specific subsections only. It is likely, however, that pet owners with exceptional interest in pet health and wellness would be most likely to voluntarily participate in the study. This could affect the results in different ways. Firstly, pet owners with specific interest in their pet’s health and wellness may be highly perceptive and aware of conditions affecting their pet, resulting in their pet being presented to their veterinarian more often. This could either prevent health disorders by implementing appropriate prevention strategies or result in earlier diagnoses. These two potential outcomes would have opposing effects on the number of health disorders reported as prevention would decrease the number of heath disorders occurring while timely diagnosis could increase the number of health disorders reported. By collecting health data reported by cat owners and not health professionals, objective evaluation of cat health could not be performed. Collection of data from veterinary practitioners has limitations as well, since less than half of cat owners present their cats to a veterinarian on a regular basis [26], and many veterinary visits are as a result of a health disorder [49]. This could result in overestimation of health disorders. Utilising a survey of pet owners, if representative of the general pet-owning population, should better represent owners of healthy pets as well as pets with health disorders. This was the goal of the study presented here. With respect to the methodology employed, the sampling strategy likely targeted pet owners with particular interest in health and wellness and it is likely that pet owners with particular interest in unconventional diets were included as it has been demonstrated that pet owners feeding unconventional diets have a strong interest in pet health and wellness [24]. This would potentially bias the results to include a higher proportion of pets fed unconventional diets. As unconventional diets have been suggested to increase risk of health disorders, this may have resulted in reporting of more health disorders than would be expected in a general population of pets. In order to compensate for this, the questionnaire was not only advertised online, but also to customers of pet retail stores, with the expectation that many of the customers would purchase commercial pet foods from these stores and thus be representative of pet owners feeding conventional foods. However, other commercial establishments where pet foods are sold, such as grocery stores or “big box” stores, were not targeted for survey advertising, which could impact the types of respondents represented in the study. As the questionnaire was available online, the number of potential pet owners who saw the link but chose not to participate (non-respondents) was indeterminable. As such, there was no ability to evaluate the response rate, nor quantify the proportion of responses obtained through advertisement a pet stores as compared to online. This impairs interpretation of how representative the sample is of the general pet-owning population. Another limitation of survey-based studies in general is the reliance on accurate reporting and representation by the pet owner, a form of recall bias. In this case, despite careful review of the diet reported utilised to determine what type of diet the cat was fed, a potential for misclassification exists if the diet reported by the cat owner did not accurately reflect what the cat was actually being fed. This is challenging to control or compensate for and represents a limitation of the study methodology that warrants consideration. Lastly, the findings presented here represent the opinions and beliefs of cat owners, not the definitive health status of the cats, and must be interpreted as such.

## Conclusions

Owners who fed their cats PB diets had a positive perception of their cats’ health, and reported a belief of better general health, better body condition, and fewer health disorders as compared to owners who fed their cats MB diets. Furthermore, the reported lifespan of cats did not differ based on diet type. While these data are owner reported and thus warrant follow-up research involving more objective evaluations, the hypothesis that owners of cats fed a PB diet would report higher prevalence of negative health outcomes was not supported by these findings.

## Methods

The study was approved by the University of Guelph Research Ethics Board (REB # 18–07-039).

### Survey design

A questionnaire was designed by the authors using the Qualtrics (Qualtrics XM, Provo, Utah, USA) online platform. The questionnaire included questions based on previously validated survey items and was piloted by the authors before being made available to potential participants. Respondents were incentivised to participate by a random draw to obtain a gift certificate for a pet store of their choosing. Eight prizes of $25CAD gift cards were available, and participants could choose to enter the prize draw by including their email address at the end of the main questionnaire. These sensitive data were removed from the main data and stored separately until the end of the data collection period. After the winning participants were contacted, all sensitive data were deleted. The questionnaire included 36 multiple-choice, 8 short answer, 7 Likert scale (0–100), and 1 ranking questions. The dataset included responses for dogs as well as cats, dog data is presented elsewhere [62]. The questionnaire used flow logic to show questions related to cats only to participants indicating that they owned cats. Questions were designed to collect demographic information about pet owners, pet species, breed, sex, age, acquisition, lifestyle (indoor/outdoor), as well as information on pet health, clinical signs and wellness. Cat BCS was selected by the respondents based on images from the World Small Animal Veterinary Association BCS chart, randomly ordered to avoid bias [63]. For this 9-point score system, a score between 1 and 3 is considered under ideal, 5 ideal, and 7 to 9 over ideal. Faecal score (FS) was selected by the respondent based on images corresponding to the Bristol stool chart [64], randomly ordered to avoid bias. A score of 1 to 2 being abnormally hard to constipated, 3 to 4 being normal, and 5 through 7 ranging from soft to diarrhoea. To determine prevalence of health disorders, body systems and common disorders were listed, along with an “other” category, and respondents were asked to select the appropriate system and describe their cat’s specific disorder. These included: behavioural, cancer, cardiovascular (heart), dental, dermatologic (skin), diabetes, ear, endocrine, eye, GI, hyperthyroid, kidney, liver, musculoskeletal, neurologic, obesity, parasites, reproductive, seizures, trauma/injury, urinary, other. Fifteen indicators of cat wellness were adapted from previously validated survey items [65–67], and answered as seven Likert scale questions and eight on a visual sliding scale. The questionnaire is available as a supplementary file (see Additional files).

### Survey distribution

A link to the survey was distributed via email and postcards to customers of Canadian and American pet food retailers, including deliberate distribution to clients of the largest PB pet food retailer in Ontario, Canada. As well, the survey was promoted in online groups of cat owners on social media (Facebook, Inc., Menlo Park, California, USA). The survey, which was available in English only, was made accessible for 9 months, from June 2018 to March 2019. Respondents represented a convenience sample of cat owners voluntarily participating in the study.

### Survey analyses

Surveys were included for analysis for each question that was completed. Breeds were categorized into breed types based on phylogenetic similarities and historical origin [68]. Description of cat diet was collected in an open-text question and, where sufficient information was obtained, categorized based on ingredients (MB, PB) and processing (commercial heat-processed, homemade, raw) or a combination of the aforementioned (i.e.: some cats were fed more than one type of food on a regular basis). The term PB referred to a diet that contained no animal ingredients, while the term MB referred to a diet that included animal-derived ingredients. Thus, while a MB diet could include plant-derived ingredients (e.g. a kibble made from chicken and soy), a PB diet could include no animal-derived ingredients. In addition to the main diet, information was also collected regarding the feeding of treats (commercial heat-processed, raw, homemade), table foods and supplements, and categorized based on ingredients (MB or PB). Cats fed a PB diet but also given treats, table foods, snacks and/or supplements containing animal-derived products, were categorized as PB+MB. All cats fed a MB diet were included in the MB category, even if their diet included PB treats, snacks and/or supplements. Due to the likelihood of eating prey even if not reportedly known to hunt [69–71], cats fed PB diets with unrestricted outdoor access or ability to hunt, were added to the PB+MB category, which was then re-classified as PB+MB/H for comparative analyses. For comparisons of pet food purchasing motivations (Table 5) and use of nutrition resources (Table 6) comparison was made between cats fed PB, PB+MB, and MB. For comparison between health outcomes (Tables 2, 3, Figs. 1 and 2), consideration of ability to hunted prey (PB+MB/H) was included as this may have an effect on cat health as compared to a strictly PB diet. Cat wellness indices measured by Likert scale were reported directly, while sliding scale data was translated by the survey software into a value reported ranging from 0 to 100. Cat health disorders were offered in multiple-choice questions, with the option of ‘other’ for input by the respondent. Owner-reported ‘other’ health disorders were categorized by a veterinarian (SD) and included for analyses. Based on the responses, behavioural, musculoskeletal, parasites, reproductive and trauma/injury were dropped. True behavioural disorders were difficult to differentiate from issues of training or lifestyle (such as “a little too sassy”, “jealous”, “grumpy” or “claws door frames and harasses our girl cat). Reported musculoskeletal issues were difficult to interpret, with ambiguous descriptions often associated with suspected previous injuries. The parasite category was dropped as responses were typically regarding kitten worming or indicated that the cat was regularly treated with endo- or ectoparasite medications, similarly the most common reproductive disorder reported was either having kittens or desexing surgery. The trauma/injury category contained mostly anecdotes of incidents that were believed to have occurred prior to current ownership, such as “broken jaw before we adopted him”, “picked up by a bird of prey prior to rescue from the street at 6 weeks”, “one rear leg amputated from abuse” and “he sustained an injury before I got him that made his back half a bit wonky”. The remaining variables recategorized to: cardiac, dental, dermatologic (including ear infections and polyps), endocrine, GI and hepatic, lower urinary tract, neoplasia, obesity, ocular and renal.

### Statistical analyses

All analyses were performed using Stata/IC 15.1 (StataCorp, College Station, Texas, USA) statistical software package. Descriptive statistics included frequency (n) and percentage (%) presented for most data (type of pets, location and time of cat acquisition, breed, indoor/outdoor management, hunting activity, diet and supplementation, motivators for selection of cat food, resources for information about feline nutrition) [72]. Frequency and percentage were also used within category for data collected using Likert scales and for ranked data. Mean and standard deviation were presented for normally distributed data (cat age, duration of cat ownership, duration of feeding (measured in years), and lifespan of previous cats) [73]. Median and range were presented for ordinal data (BCS, FS) and count data (number of cats kept, number of health disorders per cat).

Univariate comparisons between diet categories and responses related to pet food purchasing behaviours and pet nutrition information resources were conducted using χ^2^ testing (see Tables 5 and 6). These analyses were performed when comparing between diet categories only (pet food purchasing, nutrition resources, hunting activity), without consideration of potential confounders. Age of previously owned cats at death was also compared among diet groups using Kaplan-Meier statistic [74].

Statistical models were selected based on the nature of the variable of interest – count, binary, or continuous data [75]. The relationship between number of health disorders per cat, measured as count data, and diet type was modelled using multivariate negative binomial regression. Within the model, the dependent variable was the number of health disorders per cat. Independent variables considered included cat diet (3-level categorical: PB, PB+MB/H compared to MB) and potential confounders: cat age (years), sex (2-level categorical: male compared to female), sex status (2 level categorical: intact compared to desexed), breed type (9-level categorical: domestic medium hair, domestic longhair, mixed breed, Asian, American, European, other, unknown, compared to domestic shorthair), BCS (ordinal: 3, 5, 7, 9 compared to 1) and indoor/outdoor access (3-level categorical: indoor/outdoor access, outdoors only, compared to indoor only). Variables were assessed for correlations and collinearity prior to inclusion in the final model, no collinearity correction was required. Using backward stepwise elimination to remove non-significant variables, the final model included cat age, sex and diet. Model fit was visually evaluated by assessment of distribution of the residuals.

Logistic regression models were used to assess the association between individual health disorders and diet. One model was developed for each individual health disorder as the dependent variable (cardiac, dental, dermatological, endocrinological, GI and hepatic, neoplastic, obesity, ocular, renal and urinary), measured as a binary present/absent outcome. Independent variables considered included cat diet (MB, PB, PB+MB/H) and potential confounders: cat age, sex, sex status, breed type, BCS and indoor/outdoor access. Variables were assessed for correlations and collinearity prior to inclusion in the model, no corrections for collinearity were required. Backward stepwise regression was used to eliminate non-significant variables from the final multivariate model for each health disorder. Significant independent variables kept in each multivariate model differed based on health disorder (Table 3). Though non-significant for some disorders, age was forced into the model due to the known associations between increasing age and risk of health disorders as was diet, as it was the variable of interest. Model fit was assessed by Hosmer-Lemeshow goodness-of-fit test.

Ordered logistic regression modelling was used to evaluate the relationship between owner perception of cat heath, ranked in four levels: “poor”, “fair”, “good”, and “very good”. The dependent variable was health ranking, with odds ratios reported in comparison to the referent outcome “very good”. Independent variables considered included cat diet (MB, PB, PB+MB/H), cat age, sex, sex status, breed type, BCS and indoor/outdoor access. Variables were assessed for correlations and collinearity prior to inclusion in the model, no collinearity corrections were required. Backward stepwise regression was used to eliminate non-significant variables from the final multivariate model. Significant independent variables kept in the final multivariate model were cat age, sex, BCS and diet.

For all analyses, statistical significance was set at *P* < 0.05. A-priori sample size estimations were made using data regarding prevalence of feline health disorders [48]. Considering the suggested increased risk of urinary tract diseases in cats fed PB diets [22], this health disorder was chosen for sample size estimation. Assuming the proportion of cats fed MB with urinary tract disorders was 4%, calculation of the sample size comparing two different proportions with the estimation that three times as many cats fed PB would have urinary tract disorders (12%), yielded a required sample size of 116 cats per diet category. Post-hoc power calculations were performed comparing the mean values or proportions of each variable of interest between diet groups, considering an α value of 0.95 and a β of 0.8 as the cut-off points. Power less than 80% was considered too low and represented an insufficient sample size to detect significant (*P* < 0.05) differences.

## Supplementary Information

**Additional file 1.** eSurvey wording. eSurvey questionnaire. Word document transcription of electronic survey questionnaire.

**Additional file 2 **: **Table S1.** word document containing table of demographic data of survey respondents.

## Data Availability

The datasets used during the current study are available from the corresponding author on reasonable request.

## Abbreviations

BCS: Body condition score
FS: Faecal score
GI: Gastrointestinal
MB: Meat-based
PB: Plant-based
PB+MB: Plant-based main diet with meat-based snacks or treats, or animal-derived supplements
PB+MB/H: Plant-based main diet with meat-based snacks or treats, or animal-derived supplements, and/or plant-based diet with access to outdoors or the ability to hunt
