# Supplementary material for: A cross-sectional study of owner-reported health in Canadian and American cats fed meat- and plant-based diets
Source: BMC Vet Res. 2021 Jan 28;17:53. doi: 10.1186/s12917-021-02754-8 (PMC7842014; doi:10.1186/s12917-021-02754-8)
Supplement: Supplementary file 1 — Additional file 1. eSurvey wording. eSurvey questionnaire. Word document transcription of electronic survey questionnaire. [file 12917_2021_2754_MOESM1_ESM.docx]

Survey of Cat and Dog Health and Wellbeing

Consent

Thank you for considering being a participant in this study. On the next page you will find the consent form. Please read it thoroughly and indicate whether you would like to consent to participate by clicking Yes or No. Once you have reached the end of the survey, you will have the option to be entered into a draw to win one of eight $25 gift cards to a pet supply retailer of your choice.

DOG AND DOG HEALTH AND WELLNESS SURVEY PARTICIPANT CONSENT FORM

What we will ask you to do if you agree to participate:

If you live with a cat and/or dog and are their primary caregiver, you are invited to participate in research to understand factors relating to dog and cat health and wellness as reported by their keepers. If you agree to participate, you will be asked to fill out an eSurvey, which will take approximately 15-20 minutes. The eSurvey will ask you general questions about your companion animal and will inquire into their health history.

Are there any potential risks to participation?

You do not have to respond to any questions or provide information you do not feel comfortable providing. There is no consequence to you if you do not want to complete the survey. The survey will be anonymized, which means that we will not be able to connect you to the answers after the data collection period. Please note that while every effort is made to securely store data once they have been received, confidentiality cannot be guaranteed while data are in transit over the internet. Data provided by you may NOT be withdrawn from the research project once the completed survey is submitted. You may withdraw during the survey by closing your browser. Non-identifying information may be used in publishing materials and presentations.

Are there any potential benefits to participation?

Your input will benefit the area of study, and a summary of the results will be available, once the study has been completed, at: <http://bulletin.ovc.uoguelph.ca/>

Are there any financial incentives?

If you complete the survey, you can choose to enter your email into a draw for one of five $50 gift certificates to a pet supply distributor of your choice. If you enter this draw, you will have to enter your email in order to be contacted in the event that you win. No other identifying information will be requested.

Please remember that your PARTICIPATION IS VOLUNTARY and you may decide to skip or not participate at any time.

Please print or screenshot this page for your records.

Researcher information:

Dr. Adronie Verbrugghe DVM, PhD, DECVCN, Assistant Professor

Dr. Doge Dewey DVM, PhD, Professor

Dr. Sarah Abood DVM, PhD, Assistant Professor

Dr. Sarah Dodd BVSc, MSc, PhD Student

AGREEMENT TO PARTICIPATE

Please choose Yes or No (below) indidoging your consent to participation.

1. Yes
2. No

Introduction

1. Do you live with cats or dogs?
   1. Cat(s)
   2. Dog(s)
   3. Both cat(s) and dog(s)

Cats

# General

1. How many cats do you have?
   1. 1
   2. More than 1
      1. If more than 1, please specify the numbers of cats {Open Text}
2. How old is your cat?
   1. {Open Text}
3. What is the sex of your cat?
   1. Male
   2. Female
4. Is your cat neutered (castrated / spayed)?
   1. Yes
   2. No
   3. I don’t know
5. What breed is your cat?
   1. {Open Text}
6. How long has your cat lived with you?
   1. Their whole life
   2. Other
      1. {Open Text}
7. Does your cat live indoors or outdoors? If not exclusively indoors or outdoors, please describe.
   1. Indoors only
   2. Outdoors only
   3. Mostly indoors {Open Text}
   4. Mostly outdoors {Open Text}
   5. Half indoors and half outdoors {Open Text}
8. If not indoors only:

Is your cat able to hunt for prey? Do you ever see your cat catch prey?

1. Yes my cat could hunt prey, but I have never seen them catch prey
2. Yes, my cat hunts prey and I have seen them catch prey
3. No, my cat could not hunt prey, they are not loose outside
4. Please select the image which best corresponds to your cat’s body condition:
   1.
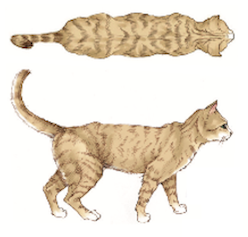

   2.
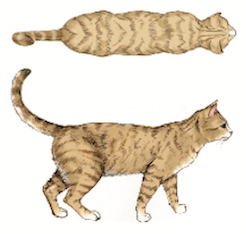

   3.
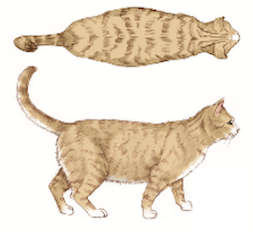

   4.
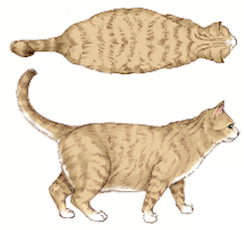


# Health

1. Please list any veterinary conditions your cat has experienced over the past year.
   1. {Open Text}
2. Please rate your cat’s current general health:
   1. Excellent
   2. Very good
   3. Good
   4. Fair
   5. Poor
3. What do you use as a source of information about cat health?
   1. Books
   2. Breeder
   3. Discussion/support groups
   4. Friends/family
   5. Internet articles written by veterinarians
   6. Internet articles written by pet owners
   7. Pet health magazines
   8. Pet health websites
   9. Pet stores
   10. Veterinarian
   11. Other
       1. {Open text}
4. How many hours per week do you spend learning about pet health related topics?
   1. < 1
   2. 1 – 10
   3. 10 – 20
   4. 20 – 30

Please answer the following questions about your cat’s health during the past 4 weeks:

1. Has your cat been ill and vomited?
   1. Not at all
   2. A little
   3. Quite a bit
2. Has your cat been inactive or had low energy?
   1. Not at all
   2. A little
   3. Quite a bit
3. Please select the image which correlates with your cat’s stool consistency.
   1.
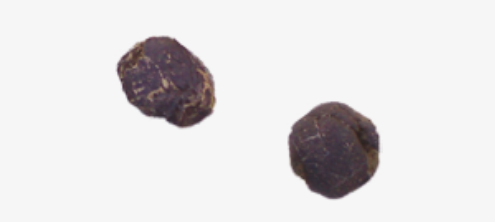

   2.
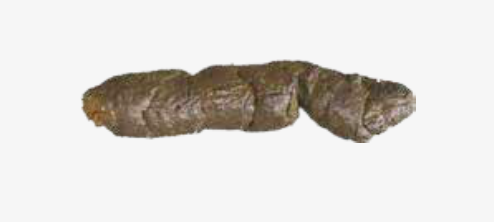

   3.
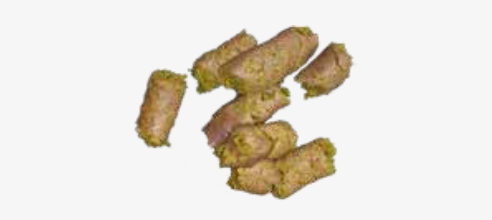

   4.
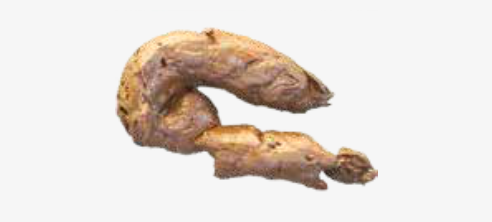

   5.
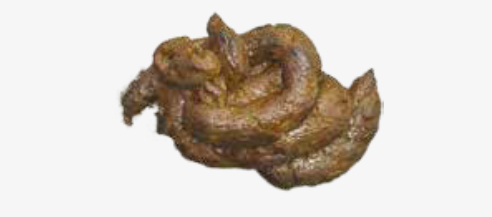

   6.
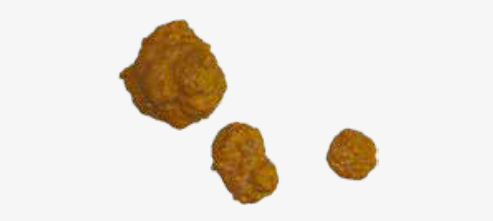

   7.
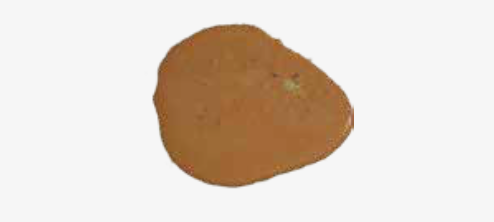

4. Has the quality of your cat’s hair coat changed?
   1. Quite worse
   2. A little worse
   3. No change
   4. A little better
   5. Quite better
5. Has your cat been grooming themselves (licking, scratching) as much as usual?
   1. A lot less
   2. A little less
   3. The same
   4. A little more
   5. A lot more
6. Has your cat been jumping (e.g. onto cat tower, furniture, counter tops) as much as usual?
   1. A lot less
   2. A little less
   3. The same
   4. A little more
   5. A lot more
7. Has your cat been drinking as much as usual?
   1. A lot less
   2. A little less
   3. The same
   4. A little more
   5. A lot more
8. Has your cat had bowel movements (passed faeces) with usual frequency?
   1. A lot less
   2. A little less
   3. The same
   4. A little more
   5. A lot more
9. Has your cat urinated with usual frequency?
   1. A lot less
   2. A little less
   3. The same
   4. A little more
   5. A lot more

# Wellbeing

### Please answer the following questions about your cat’s behaviour and wellness during the past 4 weeks:

1. Has your cat appeared happy?
   1. Not at all
   2. A little
   3. A moderate amount
   4. A great deal
2. Has your cat yowled or hissed in distress?
   1. Not at all
   2. A little
   3. A moderate amount
   4. A great deal
3. Has your cat moved away when you attempt to touch them?
   1. Not at all
   2. A little
   3. A moderate amount
   4. A great deal
4. Has your cat been affectionate towards you?
   1. Not at all
   2. A little
   3. A moderate amount
   4. A great deal
5. Has your cat been curious and shown an interest in their surroundings?
   1. Not at all
   2. A little
   3. A moderate amount
   4. A great deal
6. Has your cat been kneading (e.g. pawing laps, cushions or blankets) as usual?
   1. A lot less
   2. A little less
   3. The same
   4. A little more
   5. A lot more
7. Has your cat slept more than normal?
   1. A lot less
   2. A little less
   3. The same
   4. A little more
   5. A lot more

# Nutrition

### Please answer the following questions about your cat during the past 4 weeks:

1. How much food has your cat eaten compared to normal?
   1. A lot less
   2. A little less
   3. The same
   4. A little more
   5. A lot more
2. Has your cat’s weight changed?
   1. A lot less
   2. A little less
   3. The same
   4. A little more
   5. A lot more
3. What do you use as a source of information about cat nutrition?
   1. Book
   2. Breeder
   3. Discussion/support group
   4. Friends/family
   5. Internet
   6. Pet Store
   7. Veterinarian
   8. Other
      1. {Open text}
4. What factors do you consider important when selecting food for your cat? (select all that apply)
   1. Convenience to feed
   2. Convenience to purchase
   3. Hair ball treatment
   4. Homemade
   5. Human-grade ingredients
   6. Specific ingredients
      - 1. Presence of:
        2. Lack of:
   7. Natural/organic/holistic
   8. Palatability
   9. Plant-based/vegan
   10. Price/value
   11. Raw meat-based
   12. Skin/coat health
   13. Stool odor
   14. Stool quality
   15. Veterinary therapeutic diet prescribed for specific health condition
   16. Other
       1. {Open text}
5. Please rank the following factors in order of importance, with 1 being most important
   1. __(Choices carried over from previous question)
6. Please completely describe your cat’s diet. Include treats, snacks, table scraps or other ‘human food’, supplements, and any other sources of nutrition

(e.g. Brand “X” kibble free choice and Brand “Y” canned for breakfast and dinner, plus fish oil supplement and dental treats daily)

- 1. {Open text}

1. Has your cat been fed that type of diet for as long as you’ve had them?
   1. Yes
   2. No
      1. How long have they been fed that type of diet?
         1. {Open text}
      2. Why did you change the type of diet?
         1. {Open text}
      3. Please describe any changes you have noticed in the health or wellbeing of your cat since changing to that type of diet?
         1. {Open text}
2. Have you had previous cats, and would you be willing to answer a brief series of questions regarding them?
   1. If yes:
      1. Please indicate the age(s) your previous cat(s) lived to:
         1. {open text}
      2. What diet was/were your previous cat(s) fed?
         1. {open text}
3. Would you like to submit answers for your dog as well?
   1. Yes
   2. No

Dogs

# General

1. How many dogs do you have?
   1. 1
   2. More than 1
      1. If more than 1, please specify the numbers of dogs {Open Text}
2. How old is your dog?
   1. {Open Text}
3. What is the sex of your dog?
   1. Male
   2. Female
4. Is your dog neutered (castrated / spayed)?
   1. Yes
   2. No
   3. I don’t know
5. What breed is your dog?
   1. {Open Text}
6. How long has your dog lived with you?
   1. Their whole life
   2. Other
      1. {Open Text}
7. Does your dog live indoors or outdoors? If not exclusively indoors or outdoors, please describe.
   1. Indoors only
   2. Outdoors only
   3. Mostly indoors {Open Text}
   4. Mostly outdoors {Open Text}
   5. Half indoors and half outdoors {Open Text}
8. If not indoors only:

Is your dog able to hunt for prey? Do you ever see your dog catch prey?

1. Yes my dog could hunt prey, but I have never seen them catch prey
2. Yes, my dog hunts prey and I have seen them catch prey
3. No, my dog could not hunt prey, they are not loose outside
4. Please select the image which best corresponds to your dog’s body condition:
   1.
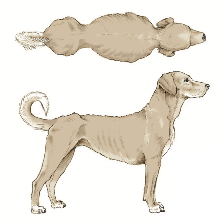

   2.
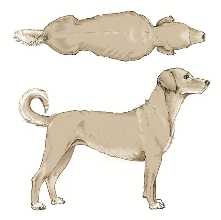

   3.
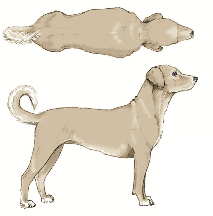

   4.
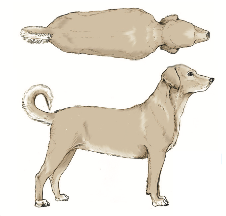

   5.
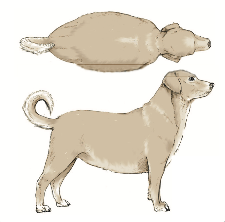


# Health

1. Please list any veterinary conditions your dog has experienced over the past year.
   1. {Open Text}
2. Please rate your dog’s current general health:
   1. Excellent
   2. Very good
   3. Good
   4. Fair
   5. Poor
3. What do you use as a source of information about dog health?
   1. Books
   2. Breeder
   3. Discussion/support groups
   4. Friends/family
   5. Internet articles written by veterinarians
   6. Internet articles written by pet owners
   7. Pet health magazines
   8. Pet health websites
   9. Pet stores
   10. Veterinarian
   11. Other
       1. {Open text}

Please answer the following questions about your dog’s health during the past 4 weeks:

1. Has your dog been ill and vomited?
   1. Not at all
   2. A little
   3. Quite a bit
2. Has your dog been inactive or had low energy?
   1. Not at all
   2. A little
   3. Quite a bit
3. Please select the image which correlates with your dog’s stool consistency.
   1.
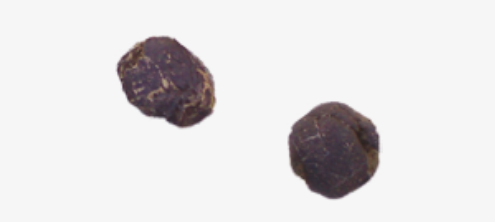

   2.
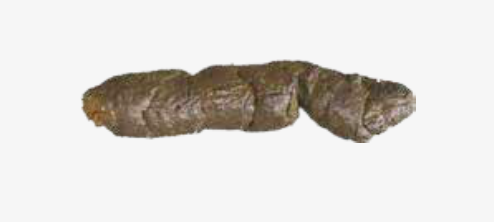

   3.
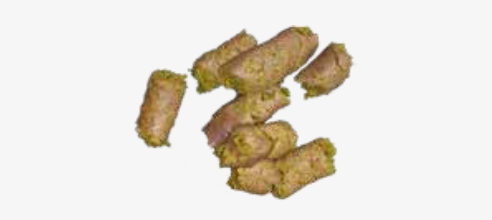

   4.
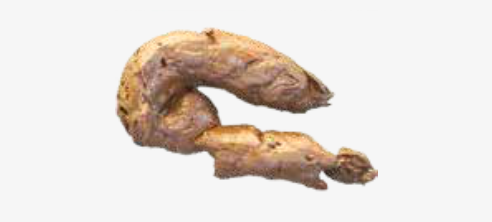

   5.
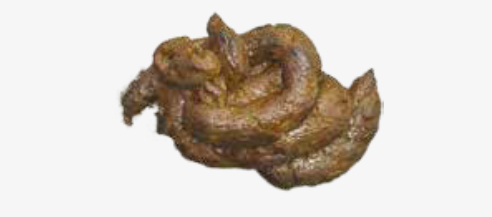

   6.
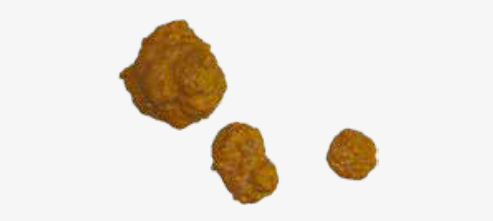

   7.
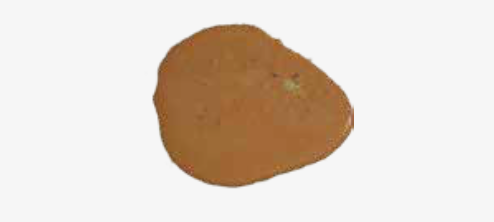

4. Has the quality of your dog’s hair coat changed?
   1. Quite worse
   2. A little worse
   3. No change
   4. A little better
   5. Quite better
5. Has your dog been slower to move around or get up after lying down?
   1. Not at all
   2. A little less
   3. Quite a bit
6. Has your dog been grooming (licking, chewing, scratching) themselves as much as usual?
   1. A lot less
   2. A little less
   3. The same
   4. A little more
   5. A lot more
7. Has your dog been easily going up or down stairs and/or jumping (such as onto a couch or into/out of a vehicle) as much as usual?
   1. A lot less
   2. A little less
   3. The same
   4. A little more
   5. A lot more
8. Has your dog been drinking as much as usual?
   1. A lot less
   2. A little less
   3. The same
   4. A little more
   5. A lot more
9. Has your dog had bowel movements (passed faeces) with usual frequency?
   1. A lot less
   2. A little less
   3. The same
   4. A little more
   5. A lot more
10. Has your dog urinated as usual?
    1. A lot less
    2. A little less
    3. The same
    4. A little more
    5. A lot more

# Wellbeing

### Please answer the following questions about your dog’s behaviour and wellness during the past 4 weeks:

1. Has your dog appeared happy?
   1. Not at all
   2. A little
   3. A moderate amount
   4. A great deal
2. Has your dog vocalized in distress (eg: howl, bark, yelp, whine)?
   1. Not at all
   2. A little
   3. A moderate amount
   4. A great deal
3. Has your dog moved away when you attempt to touch them?
   1. Not at all
   2. A little
   3. A moderate amount
   4. A great deal
4. Has your dog been affectionate towards you?
   1. Not at all
   2. A little
   3. A moderate amount
   4. A great deal
5. Has your dog been curious and shown an interest in their surroundings?
   1. Not at all
   2. A little
   3. A moderate amount
   4. A great deal
6. Has your dog been playing (e.g. chewing on toys, fetching a ball, playing with other dogs) as usual?
   1. A lot less
   2. A little less
   3. The same
   4. A little more
   5. A lot more
7. Has your dog slept as much as usual?
   1. A lot less
   2. A little less
   3. The same
   4. A little more
   5. A lot more

# Nutrition

### Please answer the following questions about your dog during the past 4 weeks:

1. What has your dog’s appetite been like?
   1. A lot less
   2. A little less
   3. The same
   4. A little more
   5. A lot more
2. Has your dog’s weight changed?
   1. A lot less
   2. A little less
   3. The same
   4. A little more
   5. A lot more
3. What do you use as a source of information about dog nutrition?
   1. Book
   2. Breeder
   3. Discussion/support group
   4. Friends/family
   5. Internet
   6. Pet Store
   7. Veterinarian
   8. Other
      1. {Open text}
4. What factors do you consider important when selecting food for your dog? (select all that apply)
   1. Convenience to feed
   2. Convenience to purchase
   3. Hair ball treatment
   4. Homemade
   5. Human-grade ingredients
   6. Specific ingredients
      - 1. Presence of:
        2. Lack of:
   7. Natural/organic/holistic
   8. Palatability
   9. Plant-based/vegan
   10. Price/value
   11. Raw meat-based
   12. Skin/coat health
   13. Stool odor
   14. Stool quality
   15. Veterinary therapeutic diet prescribed for specific health condition
   16. Other
       1. {Open text}
5. Please rank the following factors in order of importance, with 1 being most important
   1. __(Choices carried over from previous question)
6. Please completely describe your dog’s diet. Include treats, snacks, table scraps or other ‘human food’, supplements, and any other sources of nutrition

(e.g. Brand “X” kibble free choice and Brand “Y” canned for breakfast and dinner, plus fish oil supplement and dental treats daily)

- 1. {Open text}

1. Has your dog been fed that type of diet for as long as you’ve had them?
   1. Yes
   2. No
      1. How long have they been fed that type of diet?
         1. {Open text}
      2. Why did you change the type of diet?
         1. {Open text}
      3. Please describe any changes you have noticed in the health or wellbeing of your dog since changing to that type of diet?
         1. {Open text}
2. Have you had previous dogs, and would you be willing to answer a brief series of questions regarding them?
   1. If yes:
      1. Please indicate the age(s) your previous dog(s) lived to:
         1. {open text}
      2. What diet was/were your previous dog(s) fed?
         1. {open text}
