# Supplementary material for: A cross-sectional study of owner-reported health in Canadian and American cats fed meat- and plant-based diets
Source: BMC Vet Res. 2021 Jan 28;17:53. doi: 10.1186/s12917-021-02754-8 (PMC7842014; doi:10.1186/s12917-021-02754-8)
Supplement: Supplementary file 2 — Additional file 2 : Table S1. word document containing table of demographic data of survey respondents. [file 12917_2021_2754_MOESM2_ESM.docx]

**Supplementary Table S1.** Demographic data of pet owners participating in the “Pet Health and Wellbeing” survey.

| Respondent demographic | | n = 1325 | % |
| --- | --- | --- | --- |
| Country | Canada | 859 | 65 |
|  | United States of America | 466 | 35 |
| Gender | Female | 1131 | 86 |
|  | Male | 158 | 12 |
|  | Prefer not to disclose | 31 | 2 |
| Age | Less than 18 years | 10 | 1 |
|  | 18 – 24 | 122 | 10 |
|  | 25 – 34 | 328 | 25 |
|  | 35 – 44 | 257 | 19 |
|  | 45 – 54 | 292 | 22 |
|  | 55 – 64 | 202 | 15 |
|  | 65 – 74 | 83 | 6 |
|  | 75 – 84 | 8 | 1 |
|  | Greater than 85 years | 21 | 2 |
